# Fossil echinoid (Echinoidea, Echinodermata) diversity of the Early Cretaceous (Hauterivian) in the Paris Basin (France)

**DOI:** 10.3897/zookeys.325.5085

**Published:** 2013-08-20

**Authors:** Sophie Benetti, Thomas Saucède, Bruno David

**Affiliations:** 1 UMR CNRS 6282 Biogéosciences, Université de Bourgogne, 6 boulevard Gabriel, 21000, Dijon, France

**Keywords:** Echinoids, Hauterivian, Early Cretaceous, Paris Basin, France, Calcaires à Spatangues Formation

## Abstract

This dataset inventories occurrence records of fossil echinoid specimens collected in the Calcaires à Spatangues Formation (CSF) that crops out in the southeast of the Paris Basin (France), and is dated from the *Acanthodiscus radiatus* chronozone (*ca.* 132 Ma, early Hauterivian, Early Cretaceous). Fossil richness and abundance of the CSF has attracted the attention of palaeontologists since the middle of the nineteenth century. This dataset compiles occurrence data (referenced by locality names and geographic coordinates with decimal numbers) of fossil echinoids both collated from the literature published over a century and a half, and completed by data from collection specimens. The dataset also gives information on taxonomy (from species to order and higher taxonomic levels), which has been checked for reliability and consistency. It compiles a total of 628 georeferenced occurrence data of 26 echinoid species represented by 22 genera, 14 families, and 9 orders.

## Introduction

The Calcaires à Spatangues Formation (CSF) consists of shallow marine sediments deposited in the southeast of the Paris Basin (France) during the very Early Cretaceous (early Hauterivian, *Acanthodiscus radiatus* chronozone) about 132 million years ago, at the maximum of a second order sea level rise (Bulot et al. 2000; Courtinat et al. 2006; Bodin et al. 2009). Preserved deposits of near-shore and shallow marine environments are not common in Western Europe for that time-interval where deep-sea basin and deep shelf sediments predominated (Canérot and Cugny 1982; Rat et al. 1987; Schootbrugge et al. 2000). Deposits of the CSF yield a diversified, speciose and locally abundant fossil fauna, essentially composed of benthic invertebrates among which echinoids are common and locally very abundant (Cornuel 1841; Rat et al. 1987; Courtinat et al. 2006). In that respect, the CSF is a window on the little known benthic communities that thrived in shallow marine environments in the Early Cretaceous.

Fossil richness of the CSF has attracted the attention of palaeontologists since the middle of the nineteenth century (Cotteau 1857–1878, 1862–1867; Valette 1908; Corroy 1925; Rat et al. 1987; Walter 1996; Saucède et al. 2012). Fifty-four echinoid species were described in the CSF in all, half of them (26 species) based on type specimens collected in the CSF. However, many nominal species are geographically restricted and morphologically little differentiated. Of the 54 echinoid species ever described, Saucède et al. (2012) recognized only 26 species that belong to 16 different families, among which regular (13 species) and irregular (13 species) echinoids are represented in equal proportion. This still represents a high level of fossil echinoid diversity for that time-period, which can be explained by a putative high beta-diversity due to the numerous microhabitats present in shallow marine environments at that time and by the richness of cassiduloid echinoids, the group being particularly well-diversified in coarse sediment environments in the Early Cretaceous (Kier 1962).

## Project details

**Project title:** Inventory of the fossil echinoid diversity of the Early Cretaceous (Hauterivian) in the Paris Basin (France).

**Personnel:** Sophie Benetti (data manager, data publisher), Thomas Saucède (collection identifier, data collector, data manager, data publisher), Bruno David (data collector, data manager)

**Funding sources:** BioME team, UMR CNRS 6282 Biogéosciences, Université de Bourgogne.

**Figure 1.**
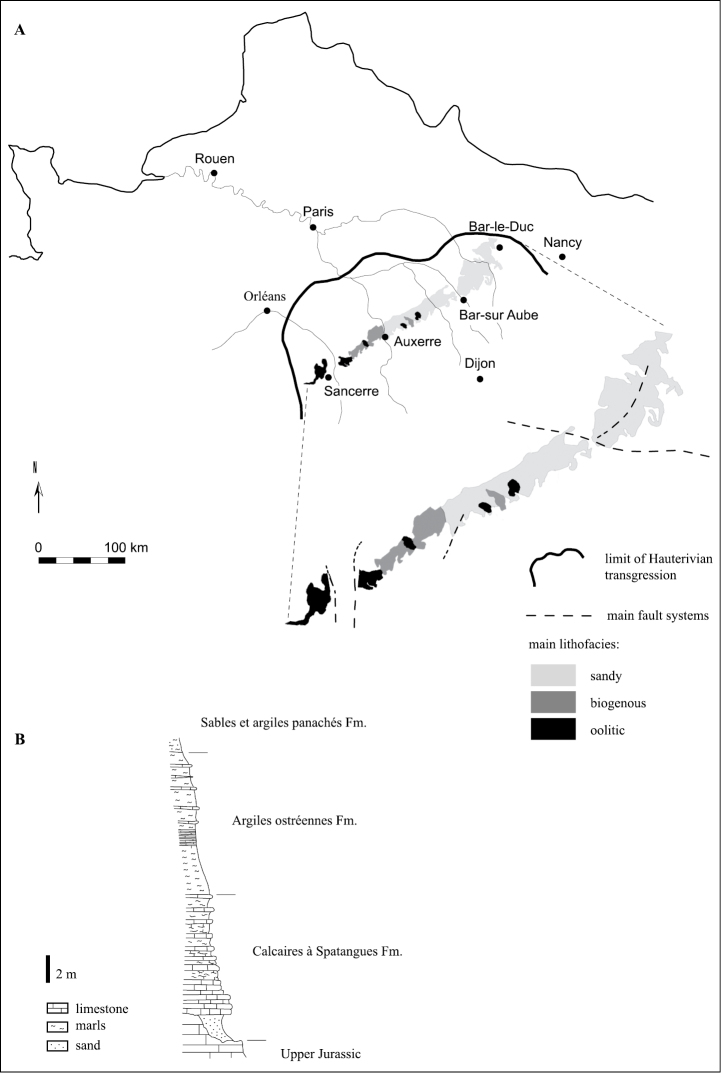
Geographic, geologic, and stratigraphic settings. A. Map showing the area of the Paris Basin (France) where the Calcaires à Spatangues Fm. crops out (modified after Courtinat et al. 2006 and Saucède et al. 2012). Distribution of main lithofacies as defined by Rat et al. (1987). B. Calcaires à Spatangues Fm. section at Lantages 48°08'N, 4°24'E (modified after Rat et al. 1987).

**Study area description.** This dataset inventories occurrence records of fossil echinoid specimens collected in the Calcaires à Spatangues Formation (CSF) that crops out in the southeast of the Paris Basin (France), from the town of Bar-le-Duc in the northeast to Sancerre in the southwest (Fig. 1A). The CSF is dated from the *Acanthodiscus radiatus* chronozone (*ca.* 132 Ma, early Hauterivian, Early Cretaceous) according to the cephalopods collected: *Acanthodiscus radiatus* (Bruguière, 1789), *Leopoldia leopoldina* group (d’Orbigny, 1841), and *Cymatoceras pseudoelegans* (d’Orbigny, 1840) (Cornuel 1841; Mégnien and Mégnien 1980; Magniez-Jannin 1984; Rat et al. 1987; Reboulet et al. 2009). The CSF is composed of limestone and clay deposits (Fig. 1B), only a few meter thick (*ca.* 0.5 to 8 m) with dominant bioclastic lithofacies (Rat et al. 1987). The average palaeo-depth was moderate (approximately few meters to *ca.* 20–30 m) based on dinoflagellates, foraminifer and bryozoan assemblages, and the overall sedimentation rate was low as suggested by the abundance of worn ferruginous bioclasts and ooliths (Rat et al. 1987; Walter 1996; Courtinat et al. 2006).

**Design description.** This dataset compiles occurrence data (all data are referenced by locality names and georeferenced WGS1984) of fossil echinoids collated from the literature published over a century and a half, from 1857 to 2012, by Cotteau (1857–1878; 1862–1867), Valette (1908), Corroy (1925), Rat et al. (1987), and Saucède et al. (2012). The dataset was completed by data from collection specimens housed at the department of Geology of Université de Bourgogne (Dijon, France), specimens sampled in the field by J Houdard, A Valette, B David, and P Robert, at the Muséum national d’Histoire naturelle (Paris, France), specimens sampled by J Lambert, and at the department of Geosciences of Université de Rennes 1 (Rennes, France), specimens sampled by P Courville. The dataset also gives information on taxonomy (from species to order and higher taxonomic levels). Systematics was reviewed and homogeneized by T Saucède for taxonomic relevance (Saucède et al. 2012).

## Taxonomic coverage

**General taxonomic coverage description:** fossil regular and irregular echinoids (Echinodermata: Echinoidea) of the Calcaires à Spatangues Formation represented by 26 species, 22 genera, 14 families, and 9 orders.

## Taxonomic ranks

**Kingdom**: Animalia

**Phylum**: Echinodermata

**Class**: Echinoidea Leske, 1778

**Orders:** Arbacioida Gregory, 1900; Cassiduloida L. Agassiz & Desor, 1847; Cidaroida Claus, 1880; Holasteroida Durham & Melville, 1957; Holectypoida Duncan, 1889; Pedinoida Mortensen, 1939; Phymosomatoida Mortensen, 1904; Salenioida Delage & Herouard, 1903; Spatangoida L. Agassiz, 1840.

**Families:** Cidaridae Gray, 1825; Hemicidaridae Wright, 1857; Emiratiidae Ali, 1990; Stomechinidae Pomel, 1883; Acropeltidae Lambert & Thiéry, 1914; Arbaciidae Gray, 1855; Saleniidae L. Agassiz, 1838; Pedinidae Pomel, 1883; Holectypidae Lambert, 1899; Conulidae Lambert, 1911; Clypeidae Lambert, 1898; Pygaulidae Lambert, 1905; Nucleolitidae Agassiz & Desor, 1847; Toxasteridae Lambert, 1920.

**Genera:** *Goniopygus* Agassiz, 1838; *Codiopsis* Agassiz, 1840; *Disaster* Agassiz, 1836; *Pseudocidaris* Etallon, 1859; *Pygurus* Agassiz, 1839; *Clypeopygus* d’Orbigny, 1856; *Nucleolites* Lamarck, 1801; *Phyllobrissus* Cotteau, 1859; *Pygorhynchus* Agassiz, 1839; *Plagiochasma* Pomel, 1883; *Plegiocidaris* Pomel, 1883; *Salvaster* Saucède, Dudicourt & Courville, 2012; *Pseudholaster* Pomel, 1883; *Globator* Agassiz, 1840; *Coenholectypus* Pomel, 1883; *Pygolampas* Saucède, Dudicourt & Courville, 2012; *Hemipedina* Wright, 1855; *Loriolia* Neumayr, 1881; *Tetragramma* Agassiz, 1840; *Stomechinus* Desor, 1856; *Hyposalenia* Desor, 1856; *Toxaster* Agassiz, 1840.

**Species:** *Plegiocidaris salviensis* (Cotteau, 1851); *Plegiocidaris lardyi* (Desor, 1855); *Plegiocidaris friburgensis* (de Loriol, 1873); *Plegiocidaris muricata* (Roemer, 1836); *Pseudocidaris clunifera* (Agassiz, 1836); *Loriolia rotularis* (Agassiz, 1836); *Loriolia bourgueti* (Agassiz, 1840); *Tetragramma autissiodorensis* (Cotteau, 1851); *Stomechinus fallax* (Agassiz, 1840); *Goniopygus peltatus* (Agassiz, 1836); *Codiopsis lorini* Cotteau, 1851; *Hyposalenia stellulata* (Agassiz, 1838); *Hemipedina minima* (Cotteau, 1851); *Coenholectypus macropygus* (Agassiz, 1836); *Globator incisa* (Agassiz, in Desor 1842); *Pygurus montmollini* (Agassiz, 1836); *Plagiochasma olfersii* (Agassiz, 1836); *Pygorhynchus obovatus* (Agassiz, 1836); *Nucleolites salviensis* Cotteau, 1851; *Phyllobrissus gresslyi* (Agassiz, 1839); *Clypeopygus paultrei* (Cotteau, 1851); *Pygolampas edita* Saucède, Dudicourt & Courville 2012; *Disaster subelongatus* (d’Orbigny, 1853); *Salvaster roberti* Saucède, Dudicourt & Courville 2012; *Pseudholaster intermedius* (Goldfuss, 1829); *Toxaster retusus* (Lamarck, 1816).

## Spatial coverage

### General spatial coverage

The sampling area focuses on the Calcaires à Spatangues Formation that crops out in the southeast of the Paris Basin (France) (Fig. 1A). The study area extends over the six following French departments: Cher, Nièvre, Yonne, Aube, Haute-Marne, and Meuse.

### Coordinates

47°33.00'N and 48°73.00'N Latitude; 2°75.00'E and 5°12.00'E Longitude.

## Temporal coverage

1851–1995.

## Collection description

**Collection names:** J Houdard, A Valette, B David, and P Robert collections housed at Université de Bourgogne (Dijon, France); P Courville collection housed at Université de Rennes 1 (Rennes, Dijon); J Lambert collection housed at Muséum national d’Histoire naturelle (Paris, France).

**Curatorial unit:** Géosciences, Université de Rennes 1 (Rennes, France), Geology department, Université de Bourgogne (Dijon, France), Muséum national d’Histoire Naturelle (Paris, France).

**Collection identifiers:** B David, T Saucède.

## Method

**Method step description.** Specimens were both collected in the field and consulted in public collections of Université de Rennes 1 (Rennes, France), Université de Bourgogne (Dijon, France), and Muséum national d’Histoire Naturelle (Paris, France). Identification of specimens was performed at species level based on descriptions by G Cotteau (1857–1878; 1862–1867), A Valette (1908), G Corroy (1925), P Rat et al. (1987), and T Saucède et al. (2012). Taxonomy was updated when it proved necessary following Kier (1962), Durham et al. (1966), Smith (1984), and Kroh and Smith (2010). Though paraphyletic, some family names have been used for convenience (Nucleolitidae Agassiz & Desor, 1847; Toxasteridae Lambert, 1920). The accuracy and geographic coordinates of localities where collection specimens came from was checked based on geological grounds (BRGM sources). Dubious localities were discarded.

**Study extent description.** The Calcaires à Spatangues Formation consists of shallow marine sediments that were deposited in the southeast of the Paris Basin (France) during the early Hauterivian (*Acanthodiscus radiatus* zone). These deposits are rich and diversified in a benthic fauna among which echinoids predominate. The systematic status of echinoids of the Calcaires à Spatangues Fm. was revised so as to update the list of echinoid species reported in the Formation and better assess its remarkable diversity. Of the 54 echinoid species ever described, 26 species are recognized that belong to 16 different families, among which regular (13 species) and irregular (13 species) echinoids are represented in equal proportion.

**Data resources**: the data underpinning analyses of the paper are deposited at GBIF, the Global Biodiversity Information Facility, http://ipt.pensoft.net/ipt/archive.do?r=hauterivian_echinoids_of_the_paris_basin

## Dataset

**Citation identifier:** http://ipt.pensoft.net/ipt/resource.do?r=hauterivian_echinoids_of_the_paris_basin

**Dataset description:** see design description

**Object name:** Darwin Core Archive hauterivian_echinoids_of_the_paris_basin

**Character encoding:** UTF-8

**Format name:** Darwin Core Archive format

**Format version:** 1.0

**Distribution:** http://ipt.pensoft.net/ipt/archive.do?r=hauterivian_echinoids_of_the_paris_basin

**Publication date of data:** 2013-08-13

**Language:** English

**Metadata language:** English

**Date of metadata creation:** 2013-08-13

**Hierarchy level:** Dataset

## Appendix

Occurrence records of Hauterivian echinoids of the Paris basin. (doi: 10.3897/zookeys.325.5083.app) File format: Microsoft Excel document (xls).

**Explanation note:** Table with occurrence data (referenced by locality names and geographic coordinates with decimal numbers) of fossil echinoids both collated from the literature and completed by data from collection specimens. The table also gives information on taxonomy (from species to order and higher taxonomic levels), which has been checked for reliability and consistency. It compiles a total of 628 georeferenced occurrence data of 26 echinoid species represented by 22 genera, 14 families, and 9 orders.
